# Social networks, depressive symptoms and quality of life in the elderly: Results of an intergenerational program

**DOI:** 10.1192/j.eurpsy.2021.1245

**Published:** 2021-08-13

**Authors:** A.P. Amaral, M.J. Nascimento, C. Rocha

**Affiliations:** Coimbra Health School, Polytechnic of Coimbra, Coimbra, Portugal

**Keywords:** quality of life, Elderly, Intergenerational, depressive symptoms

## Abstract

**Introduction:**

Intergenerational programs involving children and the elderly promote intergenerational interactions and can positively affect physical and mental health, and the quality of life of the elderly.

**Objectives:**

To test the effects of an intergenerational intervention in social isolation, depressive symptoms and quality of life of the elderly.

**Methods:**

This study employed a pretest-posttest design. Measures: Portuguese versions of Geriatric Depression Scale, Lubben Social Networks Scale and WHOQOL-OLD. Participants: 12 elderly, 75% females, with mean age of 80.8 years (sd=8.8) and 20 kindergarten children (65% female) with mean age of 4,1 years (sd=0.79). The intervention ran for 6 weeks, with 11 intergenerational group sessions, each range between 30 and 120 minutes. A nonparametric paired samples tests was conducted to evaluate the impact of the intervention.

**Results:**

After the intervention, when comparing elderly with and without depressive symptoms, results showed significant differences in the total value of quality of life (p=.048) and in the facets: 1) Sensory functioning, 2) Autonomy, 3) Past, present and future activities (p=.003; p=.018; p=.030, respectively). 12,5% of de elderly with depressive symptoms before the intervention no longer have depressive symptoms after the intervention. Regarding social networks, there were no significant differences (p=.576) between the mean values of the two assessments.

**Conclusions:**

The implemented intergenerational program was effective in promoting quality of live and minimized depressive symptoms. After the intervention, the number of the elderly without depressive symptoms have increased and these presented a higher quality of life. Finally, longitudinal studies with a large sample are needed to consolidate results.

